# Therapeutic Ketosis for Heart Failure: A State-of-the-Art Review

**DOI:** 10.1016/j.cardfail.2025.01.028

**Published:** 2025-03-03

**Authors:** NANDAN KODUR, CHRISTOPHER NGUYEN, W. H. WILSON TANG

**Affiliations:** 1 Cleveland Clinic Lerner College of Medicine of Case Western Reserve University, Cleveland, OH; 2 Department of Cardiovascular Medicine, Heart, Vascular and Thoracic Institute, Cleveland Clinic, Cleveland, OH; 3 Cardiovascular Innovation Research Center, Heart, Vascular and Thoracic Institute, Cleveland Clinic, Cleveland, OH

**Keywords:** Therapeutic ketosis, ketosis, ketone bodies, heart failure

## Abstract

Heart failure is characterized by an energy-deprived heart, and in recent years it has been found that the failing heart increases ketone body oxidation to meet its energy demands. Accumulating evidence suggests that this metabolic adaptation is cardioprotective, suggesting that interventions that boost blood ketone levels could aid the failing heart. Indeed, multiple small clinical trials with short-term follow up have demonstrated that supplying the failing heart with exogenous ketone bodies may improve myocardial function across various manifestations of heart failure. As such, therapeutic ketosis, which is a metabolic state in which blood ketone levels are mildly elevated, could have great potential to ameliorate heart failure. Therapeutic ketosis can be achieved endogenously via exercise or dietary practices, exogenously via supplementation with ketone bodies, or pharmacologically via treatment with a sodium-glucose cotransporter-2 inhibitor. Although ketosis-inducing practices cannot be routinely recommended to patients with heart failure at this time due to a lack of robust data regarding the long-term benefits and risks, anecdotal evidence suggests that some patients have begun to adopt ketosis-inducing practices, so it is important for clinicians to be aware of how to manage patients optimally when they are in therapeutic ketosis. In this review, we discuss myocardial ketone metabolism in heart failure, the current evidence for therapeutic ketosis in patients with heart failure, a framework to distinguish between therapeutic ketosis and the pathologic state of ketoacidosis, and practical considerations for managing patients adhering to ketosis-inducing practices.

## Lay summary

Ketosis is a natural metabolic state wherein the body uses ketone bodies instead of glucose as the primary energy source. This metabolic state can occur due to fasting, the ketogenic diet (which is a high-fat, carbohydrate-restricted dietary pattern), exercise, supplementation with exogenous ketone bodies, or even with the use of certain medications. Emerging research suggests that ketone bodies can improve cell health, reduce inflammation, and support blood flow and energy supply across various diseases, including heart failure, Alzheimer’s disease, diabetes, cancer, and kidney disease. As such, therapeutic ketosis, which is a metabolic state in which blood ketone levels are mildly elevated with normal blood acidity, potentially offers great promise for treating and managing these conditions. In heart failure specifically, ketone bodies may provide an alternative energy source for the failing heart as well as reduce inflammation and oxidative stress. Indeed, recent studies in patients with heart failure have shown that supplementation with exogenous ketone bodies (taken orally or intravenously) can improve heart function, at least transiently. Yet, despite the potential benefits of therapeutic ketosis, it is important to be aware of the distinction between the potentially beneficial state of therapeutic ketosis and the harmful and dangerous state of ketoacidosis, the latter of which occurs due to marked buildup of blood ketone levels resulting in increased blood acidity (this may especially occur in patients with diabetes, and in which case may also present with high blood sugar levels). Additionally, there are risks to ketosis-inducing practices such as the ketogenic diet, including increased blood cholesterol levels, nutritional deficiencies, and other side effects that could potentially increase the risk of heart disease. Overall, this paper reviews the current evidence for therapeutic ketosis in heart failure, while also highlighting the need for further research to better understand the long-term benefits and risks of therapeutic ketosis in patients with HF.

## Take-Home Figure.

Therapeutic ketosis should not be routinely endorsed for patients with heart failure at this time due to a lack of data regarding the long-term benefits and risks. Nevertheless, given that some patients have begun to adopt ketosis-inducing practices, providers should be aware of therapeutic ketosis and how to manage patients adhering to ketosis-inducing practices.

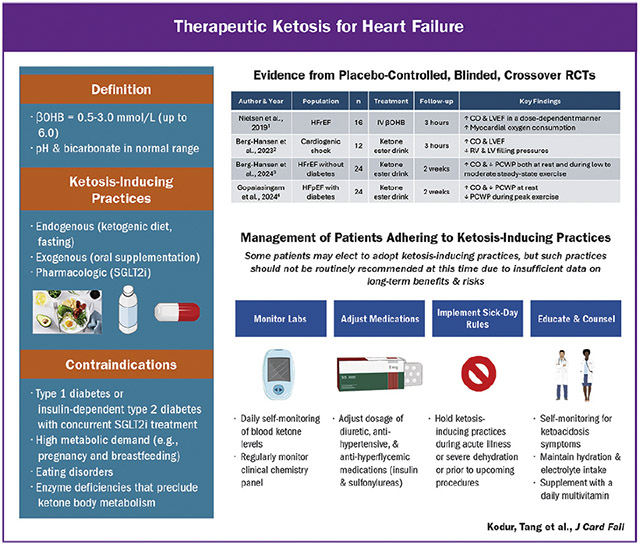

## Introduction

Heart failure (HF) affects more than 64 million adults worldwide, including over 6 million American adults, and it is associated with significant morbidity and mortality.^1^ Despite recent advances in the treatment of HF, the prognosis of this condition remains poor, with a 5-year mortality rate estimated to range from 45%–60%.^2,3^ There is therefore an urgent need for novel therapeutic interventions for HF that can add incremental benefit on top of existing medications. In recent years there has been burgeoning interest as to whether inducing the metabolic state of ketosis could be a therapeutic intervention for patients with HF, especially given that the cardioprotective benefits of sodium-glucose co-transporter 2 (SGLT2) inhibitors are posited to be in part attributable to mild ketosis.^4^ Accumulating evidence from both basic science and clinical studies suggests that ketosis could in fact aid the failing heart via numerous mechanisms, including by supplying the failing, energy-deprived heart with additional fuel.^4^ At the same time, there is anecdotal evidence suggesting that some patients have begun to adopt ketosis-inducing practices (eg, a ketogenic diet, intermittent fasting, supplementation with exogenous ketones) in an attempt to reap therapeutic benefits such as improving overall metabolic health.^5,6^ However, despite the growing adoption of therapeutic ketosis by some patients, there currently remains scant guidance on how providers can differentiate between therapeutic ketosis and the pathological state of ketoacidosis. Herein, we first discuss myocardial ketone metabolism in HF. We then provide an overview of therapeutic ketosis and discuss the current evidence for this intervention in the setting of HF. Next, we offer a framework to help providers distinguish between therapeutic ketosis and the pathological state of ketoacidosis. Finally, we conclude with some practical considerations of how clinicians can manage patients who adhere to ketosis-inducing practices.

## Methods

To devise this state-of-the-art review, we searched PubMed from its inception up to January 2025 by using a combination of the following terms: ketosis, ketone bodies, ketones, therapeutic ketosis, and heart failure.

### Increased Myocardial Ketone Metabolism in Heart Failure: A Cardioprotective Adaptation

The normal, healthy heart is metabolically flexible, and while it predominantly metabolizes fatty acids for fuel, it can adapt to physiological changes and derive energy from a variety of other fuel sources, including glucose, lactate, amino acids, and ketone bodies.^4,7^ In contrast with the normal heart, the failing heart lacks metabolic flexibility and is in turn deprived of energy.^7^ Although the different subtypes of HF manifest with varying metabolic perturbations, the energy-starvation hypothesis of HF posits that all of these different perturbations converge on the same outcome: an energy-deprived heart.^7,8^ Hence the failing heart has been dubbed an “engine out of fuel.”^9^

In response to this state of energy deprivation, the failing heart appears to increase ketone body oxidation to meet its energy demands.^4,7,8^ Evidence from clinical studies suggests that there is an increase in circulating ketone levels and, in turn, myocardial ketone uptake and oxidation in patients with HF with reduced ejection fraction (HFrEF).^4,10,12^ In a recent clinical study, for example, ketone oxidation accounted for only 6.4% of myocardial adenosine triphosphate (ATP) production in control patients but nearly tripled to 16.4% in patients with HFrEF.^13^ These findings are consistent with a previous clinical study conducted in 15 nondiabetic patients with chronic dilated nonischemic cardiomyopathy and advanced HF (mean LVEF of 17.5%), which demonstrated upregulation of the rate-limiting enzyme for myocardial ketone body oxidation (namely, succinyl-CoA:3-oxoacid-CoA transferase) along with increased myocardial ketone body use in these patients compared with 20 matched controls with nonfailing hearts.^12^ Whether myocardial ketone uptake and oxidation are also increased in patients with HF with preserved ejection fraction (HFpEF) remains less clear. A study involving 15 patients with aortic stenosis-induced myocardial hypertrophy that entailed features of HFpEF demonstrated increased myocardial uptake of ketone bodies compared with 15 control patients.^10^ On the other hand, a more recent study found that myocardial and plasma ketone levels were not elevated in 38 patients with HFpEF compared to 20 control patients, despite being elevated in 30 patients with HFrEF.^14^ Hence, while it appears that myocardial ketone oxidation is increased in HFrEF, it remains unclear whether this is also the case in HFpEF.

Mounting evidence from animal and mechanistic studies suggests that the metabolic shift toward increased myocardial ketone oxidation in HF provides the failing heart with an extra fuel source to compensate for its energy deficit, suggesting that this process is adaptive and cardioprotective.^4,8^ Indeed, it has been reported that mice lacking the myocardial enzyme needed to metabolize the ketone body beta-hydroxybutyrate (*β*OHB) experienced a greater degree of worsening HF when faced with stressors such as fasting or a pressure-overload/ischemic insult as compared with wild-type controls.^15^ Conversely, mice that retained the ability to perform myocardial ketone oxidation experienced amelioration of pathological cardiac remodeling upon increased supply of ketone bodies via the ketogenic diet.^15^ Along with being cardioprotective, under some physiological conditions increased myocardial ketone metabolism might not impair myocardial glucose or fatty-acid metabolism,^8,16^ suggesting that ketone bodies could potentially supply the failing heart with an ancillary fuel source without disrupting other metabolic processes by which the heart derives energy. Notably, increased myocardial ketone oxidation could theoretically optimize the balance between energy efficiency and production, as ketone bodies are more efficient than fatty acids with respect to ATP production per molecule of oxygen consumed, on the one hand, and generate more energy than glucose with respect to ATP production per molecule of carbon, on the other.^4,7,8^ Granted, multiple studies in both animals and humans have failed to demonstrate that ketone bodies improve myocardial efficiency.^17,18^ Nevertheless, augmenting myocardial ketone metabolism could optimize cardiac energetics by at least increasing the overall supply of fuel for the failing heart.

### Therapeutic Ketosis: An Overview

Ketosis is a metabolic state characterized by elevated blood ketone levels, which is defined by *β*OHB ≥ 0.5 mmol/L.^4^ While ketone bodies are not produced in large quantities under normal physiological settings, they are produced by the liver as an energy source for extrahepatic organs in response to stimuli that decrease glucose availability and, in turn, lower the insulin-to-glucagon ratio (eg, fasting, sleeping, exercise).^19^ Under this physiological setting, ketone bodies become a key fuel source for extrahepatic organs, such as the brain, heart and skeletal muscles.^20^ During conditions of starvation or prolonged fasting, for example, ketone bodies can account for roughly two-thirds of the brain’s energy supply.^19^ There are 3 ketone bodies that are endogenously produced via hepatic ketogenesis: acetoacetate, *β*OHB, and acetone.^19^ Under conditions that promote a low insulin-to-glucagon ratio, hepatic mitochondria increase fatty acid oxidation and, in turn, ketogenesis, producing acetoacetate. Once formed in the liver, acetoacetate can be subsequently converted into *β*OHB and acetone.^19^ Acetone cannot be metabolized and is thus exhaled or excreted via urine, whereas acetoacetate and *β*OHB serve as energy substrates for extrahepatic tissues.^19^

Therapeutic ketosis entails a metabolic state in which blood ketone levels are mildly elevated (*β*OHB = 0.5–3.0 mmol/L but sometimes reach up to 6.0 mmol/L), resulting in various salutary effects.^4,21,22^ There are three main ways of inducing therapeutic ketosis: endogenously via prolonged exercise or dietary practices such as the ketogenic diet, intermittent fasting, or time-restricted feeding; exogenously via supplementation with ketone supplements (e.g., ketone esters, ketone salts, 1,3-butanediol); and pharmacologically via SGLT2 inhibitors.^4^ While all three of these approaches induce ketosis, they have some key differences (Table 1). With regard to the mechanism by which ketosis is achieved, both endogenous and pharmacological approaches induce ketosis via a starvation-like physiological environment that lowers the insulin-to-glucagon ratio, whereas exogenous ketones bypass this metabolic reprogramming.^4,23^ In terms of the magnitude of ketosis, SGLT2 inhibitors invoke a lower degree of ketosis (<1.0 mmol/L) than endogenous or exogenous approaches (up to 3.0–6.0 mmol/L).^4,23,24^ Also, among the latter 2 approaches, exogenous ketosis likely offers greater control and fine-tuning of blood ketone levels than endogenous ketosis. On the other hand, exogenous ketones such as ketone esters boost blood ketone levels only transiently, unlike in the case of endogenous ketosis, which sustains elevated blood ketones levels over time via metabolic reprogramming.^4,23,25^ Given these differences, it is interesting to consider whether these approaches could be combined to yield synergistic effects, particularly in the case of exogenous ketones and treatment with an SGLT2 inhibitor; notably, it was recently demonstrated that concurrent treatment with both an exogenous ketone ester and an SGLT2 inhibitor can be safely implemented in patients with HF.^26^

Regardless of the means used to achieve therapeutic ketosis, this metabolic state appears to potentially offer benefit across several disease states, including HF, neurological diseases (eg, epilepsy and Alzheimer disease), diabetes, cancer, and chronic kidney disease.^4,20,21^ Emerging evidence suggests that the systemic salutary effects of ketone bodies include increased cell survival and proliferation, reduced inflammation, decreased generation of reactive oxygen species, decreased insulin resistance, enhanced immune cell function, decreased amyloid deposition, and increased blood perfusion of organs via vasodilation. Granted, it should be acknowledged that ketone bodies may have neutral or even negative effects in some situations, such as in the setting of certain tumors that develop the ability to metabolize ketone bodies.^27^

### Current Evidence for Therapeutic Ketosis in Heart Failure

**Fig. 1.**
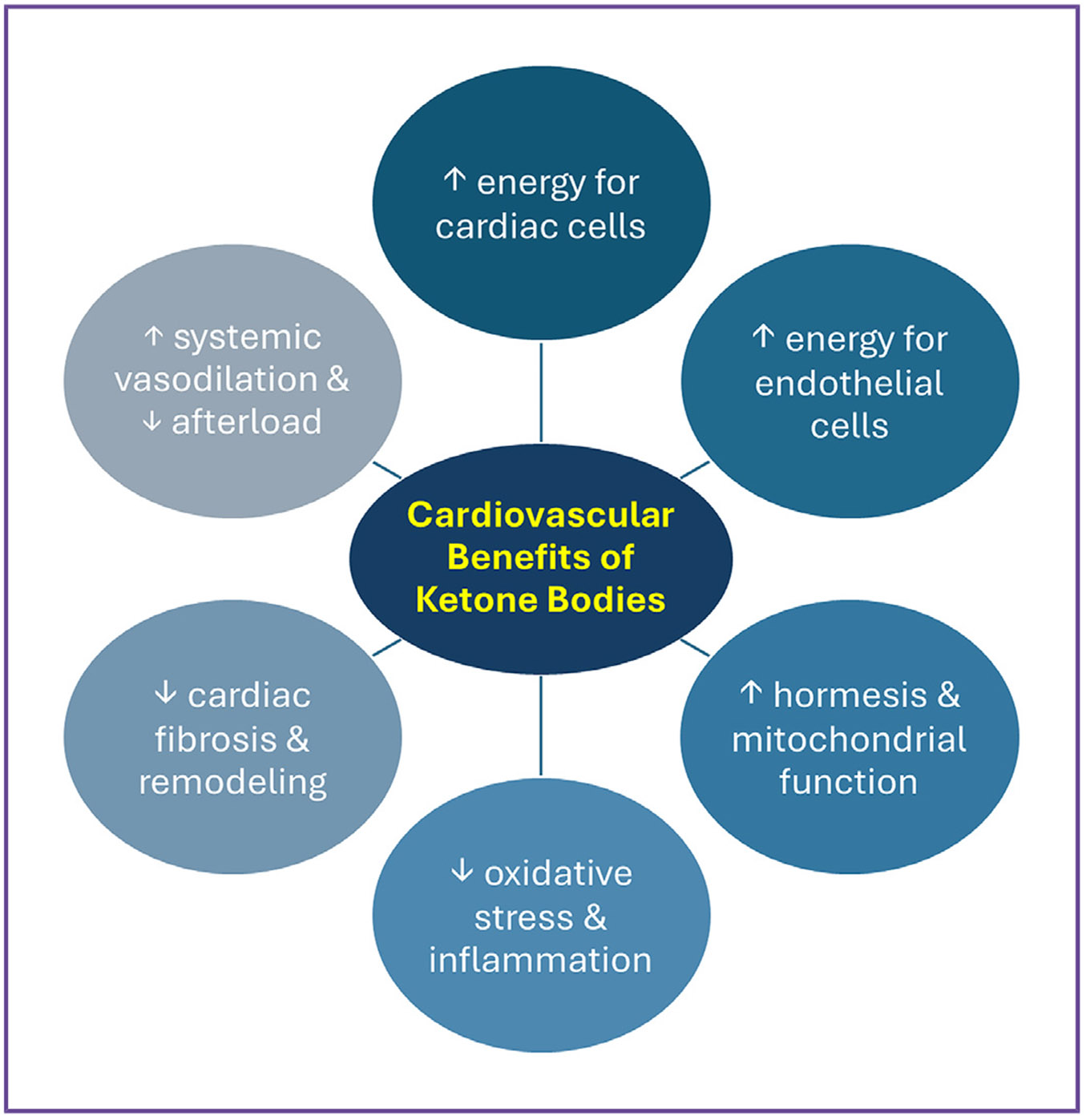
Cardiovascular benefits of ketones bodies.

Because the failing heart is deprived of energy, therapeutic ketosis might offer benefit by supplying the failing heart with an ancillary fuel source in the form of ketone bodies.^4^ This could especially be the case given that many patients with HF have inadequate caloric intake and or sarcopenia.^28,29^ In addition to boosting myocardial energetics, therapeutic ketosis could also aid the failing heart via other salutary effects (Fig. 1). Therapeutic ketosis may promote hormesis—a phenomenon in which mild stress promotes beneficial effects—and trigger cell-protective responses in cardiomyocytes and other cardiac cells.^20^ Ketone bodies may also suppress oxidative stress and inflammation in cardiac mitochondrial cells.^4^ In addition, ketone bodies may aid the failing heart indirectly by increasing proliferation of endothelial cells and attenuating pathophysiological vascular changes such as microvascular rarefaction.^30^ Relatedly, ketone bodies have been shown to promote systemic vasodilation, which may in turn reduce afterload, thereby reducing myocardial demand and increasing cardiac output.^31^ Together, these salutary effects may attenuate pathological cardiac remodeling.^4^

To date, 5 small randomized, placebo-controlled, single- or double-blind, crossover trials (n = 12–24) have demonstrated the potential myocardial benefits of supplementation with exogenous ketone bodies via either oral or intravenous delivery across various manifestations of HF—including both HFrEF and HFpEF—albeit these benefits have been measured using surrogate endpoints (eg, left ventricular ejection fraction, cardiac output) with merely modest effects observed at short follow-up durations (ranging from 3 hours to 2 weeks) (Table 2).

A randomized controlled trial (RCT) conducted in 16 patients with chronic HFrEF found that a 3-hour-long intravenous infusion of the ketone body *β*OHB transiently improved cardiac output and left ventricular ejection fraction (LVEF) in a dose-dependent manner compared with a placebo infusion.^17^ More specifically, infusing *β*OHB at a rate to achieve circulating levels of 3.3 mmol/L increased cardiac output by 2 L/min and left ventricular ejection fraction by 8%. These dose-dependent findings are consistent with previous reports demonstrating that the heart oxidizes ketone bodies in proportion to their circulating levels and, therefore, that blood ketone levels dictate the rate of myocardial ketone oxidation.^4,13^ Interestingly, it is unclear whether the improvements in cardiac output and LVEF observed in this study were primarily due to direct effects of ketone bodies on the heart resulting in increased contractility, or rather indirect effects of ketone bodies on peripheral blood vessels resulting in vasodilation and, in turn, decreased afterload, though recent evidence suggests it may be the latter.^17,18^

Recent trials investigating oral ketone esters have corroborated these results. In an RCT of 12 patients with cardiogenic shock, a single-dose enteral bolus of a ketone ester drink dosed to achieve a post-bolus peak *β*OHB level of 2.9 mmol/L resulted in improved ventricular function and tissue oxygenation compared with placebo.^32^ More recently, an RCT conducted in 24 nondiabetic patients with HFrEF found that a 14-day-long treatment regimen consisting of an oral ketone ester drink administered 4 times daily (with post-drink *β*OHB levels peaking at 2.4 mmol/L and remaining above 0.4 mmol/L for up to 4 hours) resulted in increased cardiac output as well as reduced cardiac filling pressures and volumes compared with placebo, both at rest and during exercise.^33^ Results from a follow-up analysis suggest that these benefits might have been mediated in part by cardiovascular decongestion.^34^ Notably, the investigators chose to exclude patients with diabetes from this study because of evidence suggesting that the unique metabolic state engendered by diabetes may impair myocardial ketone metabolism.^35^ Nevertheless, a separate RCT entailing 24 patients with type 2 diabetes and HFpEF found that a 2-week-long treatment regimen consisting of an oral ketone ester drink administered 4 times daily (which led to a roughly 10-fold increase in *β*OHB levels) improved cardiac output, cardiac filling pressures, and left ventricular compliance compared with placebo.^36^ While the aforementioned studies primarily investigated ketone esters, a more recent RCT sought to investigate 1,3-butanediol as a means of inducing ketosis, arguing that this approach might sustain ketosis for a longer period of time.^37^ In this RCT of 12 nondiabetic patients with HFrEF, a single-dose enteral bolus of 1,3-butanediol dosed to achieve a post-bolus peak *β*OHB level of 1.5 mmol/L resulted in improved cardiac output, stroke volume, LVEF, global longitudinal strain, and parameters of afterload.^37^ Taken together, these findings suggest that supplementation with exogenous ketone bodies could potentially offer benefit across the spectrum of HF and in the context of multiple clinical settings, warranting larger clinical trials that have longer follow-up durations and, ultimately, evaluate hard outcomes (eg, mortality, hospitalization due to HF). Indeed, the planned multicenter KETO-AHF (**Exogenous KETOne Supplements in Patients Hospitalized for Acute Heart Failure**) trial will seek to randomize 250 patients with acute HF to either a 30-day treatment regimen consisting of an exogenous ketone dietary supplement (containing 1,3-butanediol) or a taste-matched placebo, and will subsequently assess the primary hierarchical composite outcome consisting of death, HF events, change from baseline in the 6-minute walk test, and change in N-terminal pro-B-type natriuretic peptide levels (NCT06653725).

In contrast with exogenous ketone bodies, the evidence for the ketogenic diet is mixed. While the ketogenic diet has the potential to offer benefit in heart failure,^23^ some studies have found that this diet might actually result in detrimental myocardial effects, including myocardial fibrosis and senescence as well as impaired myocardial glucose oxidation.^23,38,39^ Additionally, it is thought that low-carbohydrate, high-fat dietary patterns might increase cardiovascular risk via increasing levels of low-density lipoprotein cholesterol, apolipoprotein B-containing lipoproteins, and trimethylamine N-oxide.^23,40^ Moreover, restrictive dietary patterns in patients with HF may result in unintended and unfavorable nutritional consequences such as decreased caloric intake and micronutrient deficiencies,^41^ which may especially be undesirable in patients who have concomitant sarcopenia and or cachexia. It should also be noted that many patients with HF struggle with dietary adherence,^42^ not to mention that dietary change may be a relatively low priority for these patients.^43^ Although are no published clinical trials to date investigating the myocardial effects of the ketogenic diet in patients with HF, multiple studies are currently ongoing, including a recently completed pilot study evaluating a 6-month-long ketogenic diet in 16 patients with HFpEF, with the primary endpoint being change in score on the Minnesota Living with Heart Failure Quality of Life Questionnaire (NCT04942548); an ongoing RCT seeking to randomize 90 patients with HFpEF to either a 6-month-long ketogenic diet or a low-fat diet, with the primary endpoint being change in maximal oxygen consumption (NCT06081543); and an ongoing RCT seeking to randomize 18 patients with nonischemic chronic HFrEF in a 1:1:1 fashion to a ketogenic diet, a standard diet along with supplementation with an oral ketone ester drink, or a standard diet along with supplementation with a placebo drink, with a follow-up time of 10 days and the primary endpoint being change in LVEF (NCT04921293). These studies will hopefully shed more light on the risk-benefit analysis of the ketogenic diet in the setting of HF.

In addition to exogenous ketone bodies and dietary interventions, therapeutic ketosis can also be induced pharmacologically via SGLT2 inhibitors, which have demonstrated morbidity and or mortality benefit in all subtypes of HF and are thus now considered a mainstay of treatment across the spectrum of HF.^4,44^ SGLT2 inhibitors appear to induce mild ketosis in both diabetic and nondiabetic patients alike, and it is thought that this metabolic state may partly underlie the observed cardioprotective effects, though the exact mechanisms remain uncertain.^4,45,46^ Granted, it should be acknowledged that there are some studies that have failed to demonstrate SGLT2 inhibitor-induced ketosis,^47^ though these appear to be in the minority. It is an interesting question to consider whether to whether mild ketosis is a class effect of SGLT2 inhibitors, or whether certain drugs within this class of medications are more apt to induce mild ketosis than others.

### Therapeutic Ketosis vs Ketoacidosis: Distinguishing the Good from the Bad

#### Ketoacidosis

Historically, ketosis has primarily been associated with markedly elevated ketone levels giving rise to the pathological state of ketoacidosis. The predominant manifestation of ketoacidosis is diabetic ketoacidosis (DKA), which occurs mainly in patients with uncontrolled or new-onset type 1 diabetes, though it can also occur in patients with insulin-dependent type 2 diabetes.^21^ The pathophysiology of DKA entails ketonemia, hyperglycemia, and acidemia, all stemming from insufficient amounts of insulin, which impairs cellular uptake of glucose and in turn promotes ketogenesis.^21^ DKA is diagnosed when the following criteria are met: ketonemia or ketonuria, with serum *β*OHB ≥ 3.0 mmol/L (ranging from 3.0–25.0 mmol/L); blood glucose ≥ 250 mg/dL (ranging from 250–600 mg/ dL); and blood pH < 7.3 or serum bicarbonate < 15–18 mEq/L.^21,48^ Notably, some patients with type 1 or type 2 diabetes may develop a unique form of DKA known as euglycemic DKA (eDKA), which presents with glucose < 250 mg/dL; risk factors for this condition include pregnancy, prolonged fasting, or use of an SGLT2 inhibitor with a precipitating factor (e.g., fasting).^21^ Although DKA is the most common form of ketoacidosis, there are also there are also other types of ketoacidosis, such as alcoholic ketoacidosis (AKA) and starvation ketoacidosis (SKA). AKA arises from an episode of excessive alcohol intake coupled with malnutrition, and presents with laboratory values similar to those of DKA with the exception of hypergylcemia.^19^ SKA may occur in individuals who undergo prolonged fasting, and also presents with laboratory values similar to those of DKA except for hyperglycemia.^49^

#### Distinguishing Between Therapeutic Ketosis and Ketoacidosis

Given that both therapeutic ketosis and ketoacidosis elevate blood ketone levels, but that the former is beneficial whereas the latter is detrimental, the question becomes how to clinically distinguish between these 2 metabolic states. Here, we outline a framework that can be used to differentiate therapeutic ketosis from ketoacidosis (Table 3). This framework entails assessing a patient’s entire clinical context, including magnitude of ketosis, laboratory values, clinical presentation, and medical history.

**Fig. 2.**
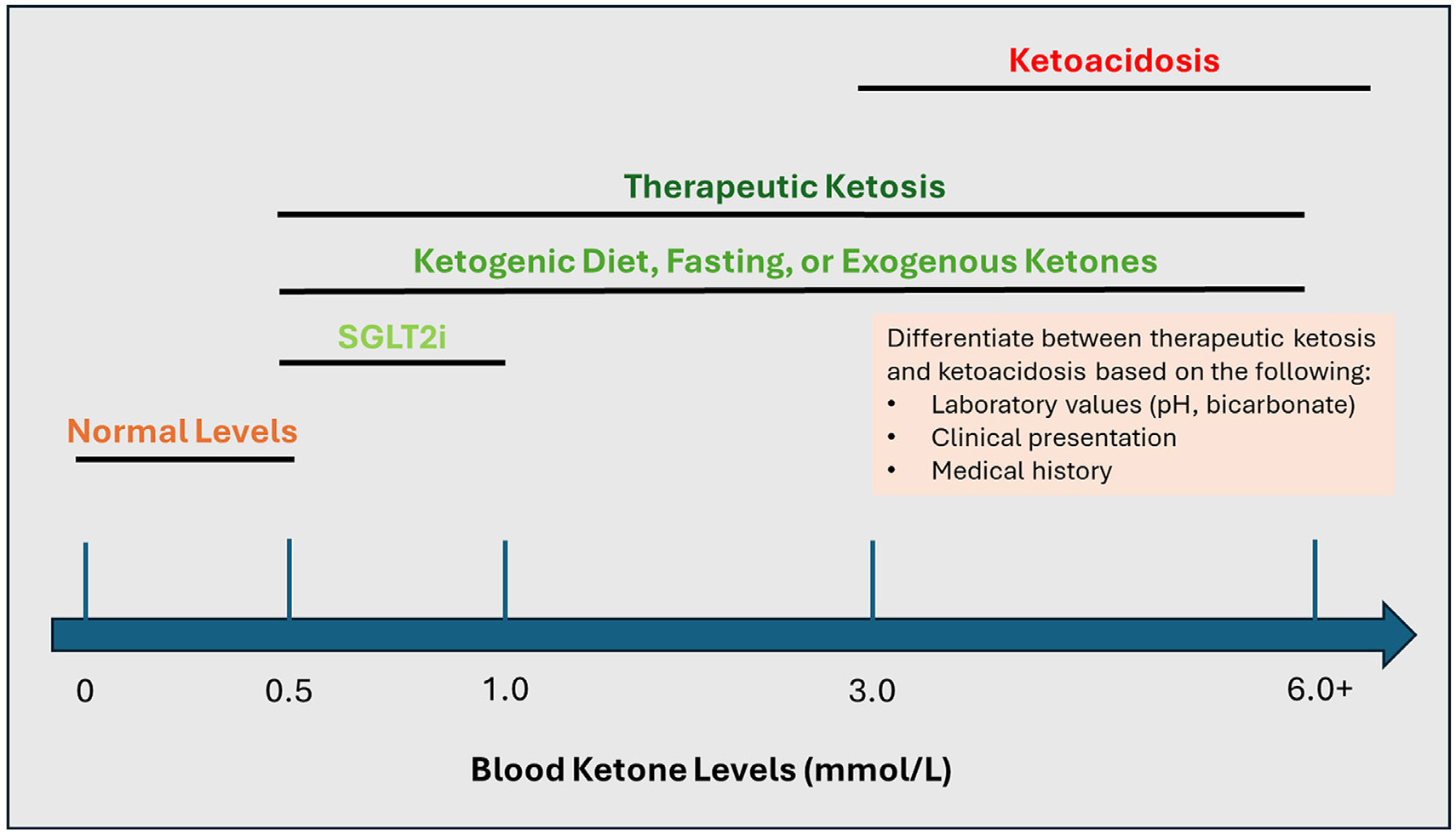
Ranges of normal, therapeutic and pathologic blood ketone levels. SGLT2i, sodium-glucose cotransporter-2 inhibitor.

Although both therapeutic ketosis and ketoacidosis entail ketosis, the magnitude of ketosis differs between these 2 metabolic states. In therapeutic ketosis, blood ketone levels are only mildly elevated, with *β*OHB = 0.5–3.0 mmol/L (but occasionally reaching up to 6.0 mmol/L),^4,50^ whereas in ketoacidosis, blood ketone levels are significantly elevated, with *β*OHB ≥ 3.0 mmol/L and reaching up to 25.0 mmol/L (Fig. 2); that said, it should be noted that ketoacidosis may present with *β*OHB < 3.0 mmol/L in the early stages of the condition, so it is important to take into account a patient’s entire clinical picture including symptoms and medical history.^50,51^ This difference in magnitude of ketosis explains why ketoacidosis is detrimental while therapeutic ketosis is not: the higher degree of ketosis in ketoacidosis engenders metabolic acidosis, whereas this does not occur in the setting of therapeutic ketosis.^51^ Hence, therapeutic ketosis and ketoacidosis can be distinguished based on blood pH and bicarbonate levels, with pH < 7.3 and bicarbonate < 15𠀓18 mEq/L in ketoacidosis but within normal ranges in therapeutic ketosis.^51^ In addition to blood ketone levels and acidity, blood glucose levels may also differ between therapeutic ketosis and ketoacidosis, though only for DKA, which presents with hyperglycemia (glucose ≥ 250 mg/dL) unlike therapeutic ketosis; notably, eDKA does not present with hyperglycemia and thus cannot be distinguished from therapeutic ketosis based on blood glucose levels.^51^

Patients’ laboratory values should be considered in the context of their clinical symptoms and medical history. In general, patients in a state of therapeutic ketosis may present with mild symptoms (eg, flu-like symptoms with initiation of the ketogenic diet, gastrointestinal discomfort, fruity breath odor), whereas patients in a state of ketoacidosis are likely to present with more severe symptoms that may be life-threatening.^4,23,52^ All forms of ketoacidosis present with symptoms of ketonemia and metabolic acidosis, including dehydration, nausea or vomiting, abdominal pain, fruity breath odor, lethargy, stupor, and the abnormal breathing pattern of rapid, deep breathing known as Kussmaul breathing.^51^ Of these symptoms, Kussmaul breathing—which is a compensatory response to metabolic acidosis—may be a key distinguishing clinical feature that is present in ketoacidosis but not in therapeutic ketosis.^51,52^ In addition to the common symptoms of ketoacidosis, the distinct types of ketoacidosis may present with unique symptoms, such as hyperglycemic symptoms in the case of DKA (eg, polydipsia, polyuria, glucosuria, blurred vision), and signs of excessive alcohol intake in the case of AKA (eg, tremulousness, agitation, abdominal pain).^52^

### Practical Considerations for Managing Patients Adhering to Ketosis-Inducing Practices

Given the current paucity of evidence regarding the long-term benefits and risks of therapeutic ketosis in the setting of HF, this intervention should not be routinely recommended to patients with heart failure at this time. That said, anecdotal evidence suggests that some patients with HF have begun to adopt ketosis-inducing practices,^5,6^ so it is important for clinicians to be aware of how to manage patients in a metabolic state of therapeutic ketosis to mitigate the risks. Patients who seek to induce therapeutic ketosis should be monitored closely and regularly, as well as counseled about the symptoms of ketoacidosis. Patients should be advised to measure ketone levels daily by using an at-home blood ketone meter that measures *β*OHB—with such tests being considered the gold standard for monitoring ketosis—to ensure that they remain within the therapeutic range; note that urine-based tests have significant measurement variability and uncertainty and are thus discouraged.^21,23^ Importantly, using a nitroprusside-based blood ketone test is strongly discouraged, as nitroprusside-based tests detect only acetoacetic acid and acetone but not *β*OHB, thereby rendering misleading results given that *β*OHB is the predominant ketone body found in states of ketoacidosis such as DKA.^51,53,54^ Monitoring blood ketone levels is especially important for patients engaging in multiple ketosis-inducing practices (eg, treatment with an SGLT2 inhibitor in the setting of a ketogenic diet, treatment with an SGLT2 inhibitor along with supplementation with exogenous ketone bodies). Although little is currently known about the interactions between these different approaches to achieving ketosis, clinicians can use a patient’s clinical symptoms and laboratory values to guide management. For most patients in a state of therapeutic ketosis, blood *β*OHB levels will range from 0.5–3.0 mmol/L, but may reach up to 6.0 mmol/L.^4,21,22^ As long as patients remain within this therapeutic range and are minimally symptomatic, clinicians can be reassured. Yet if patients develop unusual symptoms or abnormally high ketone levels, they should seek immediate medical care.

Although therapeutic ketosis does not induce a high enough degree of ketonemia to warrant concern of harm in most patients, in some high-risk patients it is possible for therapeutic ketosis to devolve into ketoacidosis.^55^ This is particularly of concern in patients with type 1 diabetes or insulin-dependent type 2 diabetes who combine treatment with an SGLT2 inhibitor with ketosis-inducing practices such as fasting or the ketogenic diet, which can result in DKA or eDKA.^22,23^ Additionally, prolonged fasting in patients with high metabolic demand (eg, pregnancy) or eating disorders can result in SKA.^49^ Hence, these patients should be strongly discouraged from initiating ketosisinducing practices, especially in the absence of close medical monitoring. Patients who develop acute illness or severe dehydration should immediately cease all ketosisinducing practices (so-called sick-day management, which includes withholding exogenous ketones and SGLT2 inhibitors). Additionally, patients who are scheduled for a medical procedure that requires prolonged fasting should temporarily discontinue ketosis-inducing practices at least a few days prior to the procedure.

For patients on a ketogenic diet, uric acid levels and a clinical chemistry panel should be obtained monthly, along with periodic monitoring of blood cholesterol levels.^23^ These patients should be particularly monitored for electrolyte and micronutrient deficiencies and should be encouraged to hydrate, maintain appropriate electrolyte intake, and supplement with a daily multivitamin.^23^ To mitigate the risk of dehydration and hypoglycemic episodes, the dosages of all diuretic, antihypertensive, and antihyperglycemic medications (especially insulin and insulin secretagogues) should be continually adjusted.^23^ Moreover, patients on a ketogenic diet should be advised to adhere to tenets of the Mediterranean diet by preferentially consuming unsaturated fats and lean meats as opposed to saturated fats and red meats, respectively, as well as opting for plant-based foods when possible.^23^ Further guidance on how to manage patients with HF who are on a ketogenic diet can be found in a recent review by Kodur et al.^23^

## Conclusions

Therapeutic ketosis potentially offers great promise for ameliorating HF (Take-Home Figure). While there is accumulating evidence for the benefits of exogenous ketone bodies, including data from multiple small clinical trials, the evidence for the ketogenic diet is currently mixed and lagging. Although there is a need for more robust evidence before ketosis-inducing practices can be routinely recommended or endorsed in the setting of HF, anecdotal evidence suggests growing adoption of ketosis-inducing practices among some patients, so it is important for clinicians to be aware of how to manage patients who are in a metabolic state of therapeutic ketosis. All patients engaged in therapeutic ketosis should be monitored closely, including daily self-monitoring of blood ketone levels, and providers should distinguish between therapeutic ketosis and the pathological state of ketoacidosis by assessing a patient’s degree of ketosis, blood pH and bicarbonate levels, clinical signs, and medical history.

Little is still known about the long-term benefits and risks of therapeutic ketosis in patients with HF. Mechanistic studies are warranted to elucidate the impact of therapeutic ketosis on metabolic remodeling, as well as to identify phenotypes of patients that might be most conducive to therapeutic benefit. Future clinical trials are needed to determine the optimal range of therapeutic ketosis, as well as the optimal dosages and durations of ketosis-inducing interventions. Such trials should also interrogate the various means of achieving therapeutic ketosis, including whether they can be combined to yield synergistic effects, such as in the case of supplementation with exogenous ketone esters and treatment with SGLT2 inhibitors. Ultimately, large-scale trials with long-term follow-up duration are warranted to establish the long-term benefits and risks of ketosis-inducing practices, including assessment of hard outcomes such as major adverse cardiovascular events. Together, these studies will inform us as to whether therapeutic ketosis can be leveraged to augment existing therapeutic regimens for HF.

## Figures and Tables

**Table 1:** Comparison of the 3 main approaches to inducing therapeutic ketosis

	Endogenous	Exogenous	Pharmacologic
Examples	Ketogenic diet Intermittent fasting or time-restricted eating Prolonged exercise	Ketone esters Ketone salts 1,3-butanediol	SGLT2 inhibitor
Mechanism	Decreased insulin-to-glucagon ratio	Direct increase in ketone levels	Decreased insulin-to-glucagon ratio
Magnitude of Ketosis (*β*OHB levels)	0.5–3.0 mmol/L (up to 6.0 mmol/L)	0.5–3.0 mmol/L (up to 6.0 mmol/L)	0.5–1.0 mmol/L
Duration of ketosis	Sustained	Transient	Sustained
Other Salient Points	Ketogenic diet may be associated with adverse effects, such as myocardial fibrosis and senescence, as well as worsening lipid profiles	Multiple small RCTs have demonstrated that exogenous ketones may improve myocardial function Supplementation with exogenous ketones allows for greater control and fine-tuning of blood ketone levels Ketone salts invoke a lower degree of ketosis than ketone esters Ketone salts may cause increased salt loads 1,3-butanediol might induce more prolonged ketosis	Mild ketosis may partly account for the cardioprotective benefits of SGLT2 inhibitors

*β*OHB, beta-hydroxybutyrate; RCT, randomized controlled trial; SGLT2, sodium-glucose cotransporter-2.

**Table 2:** Clinical trials investigating the myocardial effects of supplementation with exogenous ketone bodies in patients with heart failure

Author, Year	Design	Patient Population	Size	Treatment	Follow-Up Duration	Key Findings
Nielsen et al., 2019^17^	Randomized, placebo-controlled, crossover trial with blinding of participants	Chronic HFrEF	16	Intravenous infusion of *β*OHB at a rate to achieve circulating levels of 3.3 mmol/L	3 hours	Cardiac output increased by 2 L/min (40%), owing to an increase in stroke volume by 20 mL and an increase in heart rate by 7 beats per minute LVEF improved by an absolute increase of 8% Improvements in cardiac output and LVEF were dose dependent Myocardial oxygen consumption increased, without impairment of myocardial external energy efficiency
Berg-Hansen et al., 2023^32^	Randomized, placebo-controlled, double-blind, crossover trial	Cardiogenic shock	12	Single-dose enteral bolus of a ketone ester drink (0.5 g/kg) achieving a post-bolus peak *β*OHB level of 2.9 mmol/L	3 hours	Cardiac output and power output improved LVEF improved by 4 percentage points Right and left ventricular filling pressures were reduced Tissue oxygenation increased
Berg-Hansen et al., 2024^33^	Randomized, placebo-controlled, double-blind, cross-over trial	HFrEF without diabetes	24	Oral ketone ester drink (25 g) taken 4 times daily, with post-drink *β*OHB levels peaking at 2.4 mmol/L and remaining above 0.4 mmol/L for up to 4 hours	2 weeks	At rest, cardiac output increased by 0.3 L/min and pulmonary capillary wedge pressure decreased by 2 mm Hg During low to moderate steady-state exercise, cardiac output increased by 0.5 L/min and pulmonary capillary wedge pressure decreased by 3 mm Hg Exploratory analyses revealed an absolute increase in LVEF by 3%, reduction in N-terminal pro-B-type natriuretic peptide levels by 18%, and reduced left atrial and ventricular volumes
Gopalasingam et al., 2024^36^	Randomized, placebo-controlled, double-blind, cross-over trial	HFpEF with type 2 diabetes	24	Oral ketone ester drink (25 g) taken 4 times daily, resulting in a roughly ten-fold rise in *β*OHB levels	2 weeks	At rest, cardiac output increased by 0.2 L/min and pulmonary capillary wedge pressure decreased by 1 mm Hg During peak exercise, pulmonary capillary wedge pressure decreased by 5 mm Hg, with concomitant improvement of the pressure-flow relationship Left ventricular compliance increased
Guldbrandsen et al., 2025^37^	Randomized, placebo-controlled, cross-over trial with blinding of participants	HFrEF without diabetes	12	Single-dose enteral bolus of 1,3-butanediol (0.5 g/kg) achieving a post bolus peak *β*OHB level of 1.5 mmol/L	6 hours	Cardiac output increased by 0.9 L/min Stroke volume increased by 15 mL LVEF increased by 3 percentage points Global longitudinal strain improved Parameters of afterload decreased Free fatty acid and glucose levels decreased

*β*OHB, beta-hydroxybutyrate; HFpEF, heart failure with preserved ejection fraction; HFrEF, heart failure with reduced ejection fraction; LVEF, left ventricular ejection fraction.

**Table 3:** Framework for distinguishing between therapeutic ketosis and ketoacidosis

	Therapeutic Ketosis	Pathologic Ketosis (Ketoacidosis)			
Ketone body concentration (blood)	*β*OHB = 0.5–3.0 mmol/L (may reach up to 6.0 mmol/L)	*β;*OHB ≥3.0 mmol/L (3.0–25.0 mmol/L)			
pH and bicarbonate levels (blood)	pH within normal range Bicarbonate within normal range	pH <7.3 Bicarbonate < 15-18 mEq/L			
		DKA	eDKA	AKA	SKA
Glucose levels (blood)	Glucose within normal range	Glucose ≥250 mg/dL (250–600 mg/dL)	Glucose <250 mg/dL	Glucose low or within normal range	Glucose low or within normal range
Medical history	Adherence to a ketogenic diet Adherence to intermittent fasting or time-restricted feeding Prolonged exercise Supplementation with exogenous ketone bodies Treatment with an SGLT2 inhibitor	Type 1 diabetes that is uncontrolled or new onset	Insulin-dependent type 2 diabetes that is uncontrolled Type 2 diabetes treated with an SGLT2 inhibitor with some precipitating factor (e.g., fasting) Diabetes with prolonged fasting or pregnancy	Chronic alcohol use with an episode of excessive alcohol intake (especially with poor food and liquid intake)	Prolonged fasting, especially in patients with high metabolic demand (e.g., pregnant patients) or an eating disorder
Clinical presentation	Ketogenic diet: Flu-like symptoms for a few weeks following initiation of the diet, known as “keto flu” (e.g., fatigue, headache, abdominal pain, constipation, muscle cramps, dehydration) Ketogenic diet and exogenous ketones bodies: Gastrointestinal discomfort Fruity breath odor	Symptoms of hyperglycemia (i.e., polydipsia, polyuria, glucosuria, blurred vision) Kussmaul breathing Lethargy Stupor Fruity breath odor Nausea or vomiting Dehydration	Same symptoms as DKA but without symptoms of hyperglycemia	Same symptoms as DKA but without symptoms of hyperglycemia Signs of acute excessive alcohol intake (e.g., tremulousness, abdominal pain, agitation)	Same symptoms as DKA but without symptoms of hyperglycemia

AKA, alcoholic ketoacidosis; *β*OHB, beta-hydroxybutyrate; DKA, diabetic ketoacidosis; eDKA, euglycemic diabetic ketoacidosis; SGLT2, sodium-glucose cotransporter-2; SKA, starvation ketoacidosis.

This framework was created based on papers by Sacks et al.,^51^ Gosmanov et al.,^52^ Anderson et al.,^21^ Cashen et al.,^48^ Laffel et al.,^19^ and Cahill et al.^49^
